# Management of LVAD in Cerebral Salt‐Wasting Syndrome

**DOI:** 10.1155/crcc/7068825

**Published:** 2025-12-18

**Authors:** Kevin Emmanuel Moriles, Ryu Peter Tofts, Denny Oliveira, Bibidh Subedi, Arnaldo Lopez-Ruiz

**Affiliations:** ^1^ Department of Critical Care Medicine, AdventHealth Orlando, Orlando, Florida, USA, adventhealth.com; ^2^ Department of Graduate Medical Education, AdventHealth Orlando, Orlando, Florida, USA, adventhealth.com; ^3^ Department of Pharmacy, AdventHealth Orlando, Orlando, Florida, USA, adventhealth.com

## Abstract

The use of left ventricular assist devices (LVADs) is now commonplace in the management of nonischemic cardiomyopathy. LVADs are preload‐dependent and afterload‐sensitive, meaning rapid changes in a patient′s volume status can significantly impact device function. Cerebral salt‐wasting syndrome (CSWS), a rare cause of hyponatremia, is characterized by an acute increase in urine output and low serum sodium. Here, we present the case of a 43‐year‐old man with end‐stage heart failure supported by an LVAD, who experienced refractory low‐flow alarms due to CSWS. The patient initially presented with a new‐onset headache. Brain computed tomography (CT) scan revealed an acute subdural hematoma with midline shift, requiring emergency craniotomy, which was performed without complications. After discharge from the intensive care unit (ICU), he returned with multiple low‐flow alarms on his LVAD (flow less than 1.5 L/h), despite high mean arterial pressures and normal perfusion indexes. Laboratory evaluation showed a serum sodium of 130 mmol/L, a urine osmolality of 522 mmol/kg, a serum osmolality of 282 mmol/kg, and a urine sodium of 147 mEq/day. His urine output was 4 L/day. A diagnosis of CSWS was made. Treatment included normal saline drip for 1:1 volume replacement, daily fludrocortisone, low‐dose desmopressin, and intermittent 3% hypertonic saline boluses. Salt tablets were prescribed to maintain a serum sodium goal of 135–140 mmol/L and a urine output of 2.5–3.5 L/day. Over time, the frequency of low‐flow alarms decreased significantly, and the patient was discharged. CSWS is an unlikely cause of hyponatremia in the ICU. Aggressive fluid hydration and sodium supplementation are necessary for positive clinical outcomes. This is especially true in patients with LVADs, who are both preload‐dependent and afterload‐sensitive. Failure to rapidly treat CSWS can lead to respiratory arrest, brainstem herniation, and even death.

## 1. Introduction

The left ventricular assist device (LVAD) is now a viable long‐term option for enhancing cardiac function in advanced end‐stage heart failure. The LVAD is a surgically implanted, life‐saving device that helps the left ventricle pump blood to the body. It is indicated for patients with critical left ventricular dysfunction who continue to have severe symptoms of heart failure despite optimal guideline‐directed medical therapy (GDMT) [1]. These patients typically have a left ventricular ejection fraction (LVEF) of less than 30% and are classified as New York Heart Association (NYHA) Class IV for symptoms at rest. LVADs are used in three ways: as a bridge to transplant for patients awaiting heart transplantation, as destination therapy for patients ineligible for transplantation but needing long‐term support, and as a bridge to recovery for patients needing temporary support while the heart function recovers. The strongest evidence for LVAD use in end‐stage heart failure comes from the REMATCH trial in 2001. This trial showed that LVADs reduced all‐cause mortality compared to optimal medical therapy [2]. Over the past decade, LVAD utilization in the United States has significantly increased. There has also been a shift toward using LVADs more frequently as destination therapy rather than solely as a bridge to transplant [3]. The pump flow of an LVAD depends on the blood volume returning to the left ventricle, making these devices preload‐dependent. States that decrease preload, such as hypovolemia, can lead to decreased systemic perfusion and may cause rapid cardiogenic shock. LVADs are also afterload‐sensitive; increased afterload, as seen in worsening hypertension, raises the resistance against which the pump must work. Thus, drastic changes in a patient′s volume status have significant clinical implications for patients with LVADs. These devices require a stable, euvolemic volume status and may quickly malfunction otherwise. Patients with LVADs are also at risk of ischemic strokes. One systematic review identified 735 articles with 11,310 patients who had LVADs and found an increased risk of ischemic stroke. This study reported a median mortality rate of 31% for LVAD‐associated ischemic strokes [4]. Because of this increased risk of clots, patients with LVADs require lifelong anticoagulation, which also raises the risk of bleeding [5].

Hyponatremia is one of the most commonly encountered electrolyte derangements in the ICU. Most cases are due to the syndrome of inappropriate antidiuretic hormone secretion (SIADH), but a rare cause is cerebral salt‐wasting syndrome (CSWS). CSWS typically presents with increased urine output, rapid hypotension, and worsening hyponatremia from excessive sodium excretion. In contrast, SIADH results from inappropriate antidiuretic hormone release, causing decreased urine output and worsening hyponatremia as free water is rapidly reabsorbed. Both SIADH and CSWS can occur after acute cerebral hemorrhages, likely due to increased intracranial pressure. Their management differs: SIADH requires fluid restriction, while CSWS needs aggressive fluid supplementation. SIADH is a common cause of hyponatremia in the ICU, but CSWS, though rare, must also be considered. One study reviewed 316 patients with subarachnoid hemorrhage and hyponatremia and found that SIADH accounted for 69% of cases, and CSWS 6.5%, illustrating its rarity and significance [6]. After further literature review, there are no meta‐analyses or literature reviews on LVAD management in CSWS. One 2010 case report described a patient with a LVAD, subdural hematoma, and cerebral salt wasting. This case had elevated B‐type natriuretic peptide (BNP) without volume overload, suggesting a noncardiac cause for the BNP rise. Not only does this hint at BNP′s potential value in CSWS management, but it also highlights the limited research on this topic. This paper describes a 43‐year‐old man with end‐stage heart failure on LVAD support. He presented with a subdural hematoma and low‐flow alarms due to CSWS.

## 2. Case Presentation

**Figure 1:**
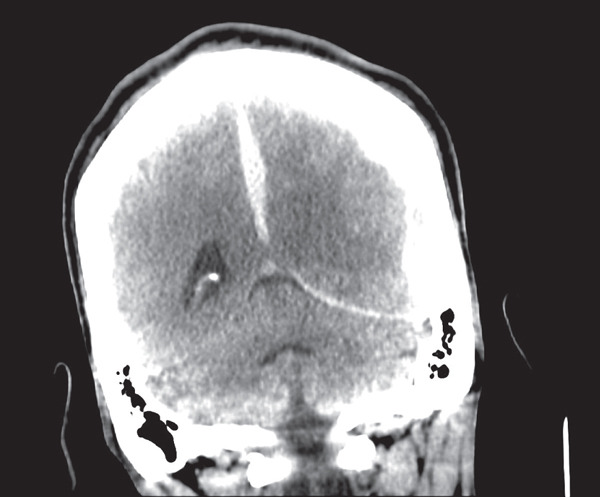
CT brain without contrast, horizontal view.

**Figure 2:**
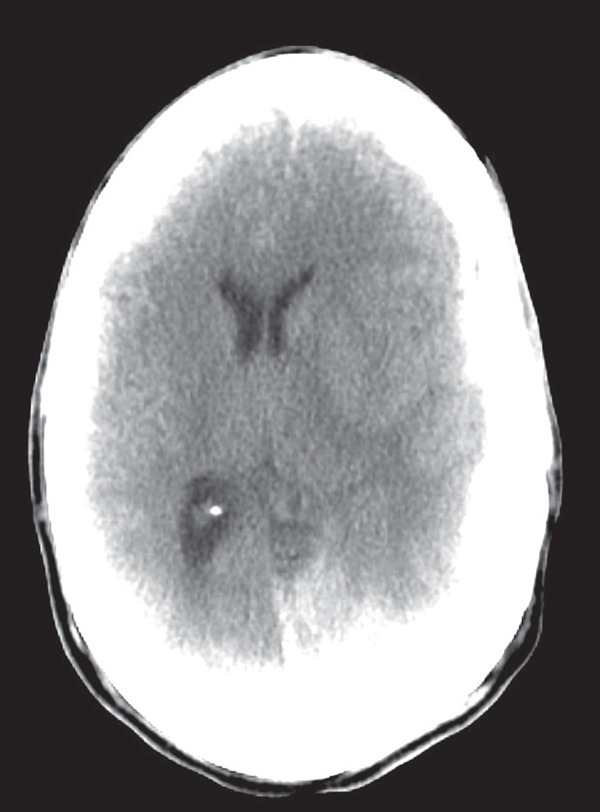
CT brain without contrast, coronal view.

The patient is a 43‐year‐old male with a past medical history of end‐stage heart failure with an LVEF of less than 15%, peripheral arterial disease, a recently inserted LVAD HEARTMATE III, and warfarin use for anticoagulation, who presented for an initial complaint of headache. The patient had an LVAD implanted approximately 1 month ago. He was seen in outpatient follow‐up 4 days before hospitalization without any medical issues. He was in his normal state of health that morning when he suddenly complained of a nonradiating headache. He quickly became unresponsive. He was brought to the emergency department, where he was immediately intubated; administered fresh frozen plasma and vitamin K, as INR was 3.1; and sent for imaging. The initial scan revealed a subdural hematoma measuring 1.4 cm with a 1.0 cm midline shift as seen in Figures 1 and 2. Because the patient was not adequately sedated, the images were motion‐degraded and had significant artifacts. This precluded adequate and deep scrutiny of the brain parenchyma and extra‐axial spaces, but the enlarging subdural hematoma was apparent. The patient underwent an emergency craniotomy with evacuation. Per the operative reports, there was a herniation in progress and two small cortical lacerations. There was a slight amount of oozing, with no evidence of arterial injury. A subgaleal drain was placed, and the patient was transferred to the ICU with no immediate complications. He was later extubated and returned to his baseline mentation with only mild generalized weakness. The drain was eventually removed, and he underwent middle meningeal artery embolization. A follow‐up brain scan revealed a chronic subdural hematoma with negligible mass effect and was deemed stable. Therefore, anticoagulation with a heparin drip was started to decrease the risk of ischemic stroke and thrombus formation within the LVAD.

He was being managed on the floor when the patient began having recurrent low‐flow alarms, where the flow rate was less than 1.5 L/h. Initial LVAD settings showed the flow at 3 L/min intermittently, speed at 5800 RPM, pulsatility index of 1.1, and power at 4.6 W. LVAD interrogation did not reveal any mechanical malfunction, and the driveline site was clean without evidence of infection. They reduced the speed to 4900 RPM to see if that would decrease the frequency of alarms. The patient continued to have persistent and numerous low‐flow alarms despite high mean arterial pressures and perfusion indexes. The patient was then transferred back to the ICU for further management. He was initiated on milrinone 0.125 mcg/kg/min and started on empiric antibiotics; his goal‐directed medical therapy was held, Lactated Ringer′s maintenance fluid was initiated, and a Swan–Ganz catheter was placed. The right heart catheterization demonstrated CVP 8 mmHg, PAP 36/4 (15) mmHg, PCWP 7 mmHg, SVR 904 dynes/sec/cm^−5^, tdCO 6.10 L/min, CI 3.24 L/min/m^2^, mixed venous 75.1%, and Pro‐BNP 248 pg/mL. Initial ICU lab work can be reviewed in Table 1. CT angiography of the chest/abdomen/pelvis did not reveal any thrombus or occlusions within the LVAD. There was no evidence of acute infection, perforation, or bleeding, especially in the area around the LVAD insertion site. A repeat CT brain scan revealed a stable subdural hemorrhage and 0.3 cm unchanged midline shift. The echocardiogram revealed no malposition of the LVAD cannulas. During this time, the patient was producing 4 L of urine a day without the use of diuretics. Table 2 shows a steady rise in urine output during his hospitalization. Therefore, urine studies were obtained that revealed a urine sodium of 147 mmol/L, a urine osmolality of 522 mOsm/kg, a serum sodium of 130 mEq/L, a serum osmolality of 282 mOsm/kg, a serum uric acid of 1.6 mg/dL, and an arginine vasopressin hormone of < 0.5 pg/mL. Urinalysis did not reveal any infection, RBCs, or proteins. Due to the elevated urine output, the decrease in serum sodium, the increase in urine sodium, and the low uric acid, he was diagnosed with CSWS. CSWS was secondary to his recent subdural hematoma, which was inducing hyponatremia and hypovolemia. Thus, the preload was not adequate for the LVAD to provide appropriate blood flow and was the etiology of the repeated low‐flow alarms.

**Table 1:** Initial ICU lab work and urine studies.

	**Values**
Complete metabolic count	
Sodium	130 mmol/L
Potassium	4.0 mmol/L
Chloride	104 mmol/L
Carbon dioxide	24 mmol/L
Anion gap	6 mmol/L
BUN	12 mg/dL
Creatinine	0.75 mg/dL
eGFR	114.8 mL/min
Glucose	103 mg/dL
BNP	248 pg/nL
Complete blood count	
White blood cell count	16.67
Red blood cell count	4.04
Hemoglobin	10.1
Hematocrit	32.3
Platelet count	283
Lactic acid	1.00
Serum and urine studies	
Serum osmolality	282 mOsm/kg
Urine osmolality	552 mOsm/kg
Sodium urine	147 mmol/L
Hematocrit	32.3
Platelet count	283

**Table 2:** Urine output and BNP trends.

24‐h intake/output/net			
Date	Intake	Output	Net
10/17	+3604 mL	−2025 mL	+1579 mL
10/18	+3961 mL	−4725 mL	−763.8 mL
10/19	+3643 mL	−2375 mL	−566 mL
10/20	+5212 mL	−6795 mL	−1582 mL
10/21	+3981 mL	−5020 mL	−1038 mL
10/22	+4000 mL	−4755 mL	−774.1 mL
10/23	+2837 mL	−2580 mL	+257 mL
10/24	+1952 mL	−4700 mL	−2747 mL
10/25	+6549 mL	−8015 mL	−1465 mL
11/14	+1630 mL	−2215 mL	−585 mL

BNP trend			
Date	Values		
10/17	248 pg/mL		
10/18	168 pg/mL		
10/19	471 pg/mL		
10/20	1007 pg/mL		
10/21	825 pg/mL		
10/22	1100 pg/mL		
10/23	758 pg/mL		
10/24	1162 pg/mL		
10/25	710 pg/mL		
11/14	267 pg/mL		

He was initially managed with scheduled fludrocortisone and intravenous fluid resuscitation. However, the low‐flow alarms persisted, as did excessive urine output. Desmopressin (DDVAP) 1 mcg intravenously daily was scheduled with boluses of 3% saline. The goal was to target a serum sodium between 135 and 140 mmol/L and a urine output of 2.5–3.5 L daily. His maintenance fluid was switched to normal saline for 1:1 supplementation at a higher rate of administration. Table 2 details the trend of his BNP as his symptoms slowly improved. After 3 weeks of treatment, targeting the parameters outlined above, the patient′s urine output eventually decreased dramatically. The patient was weaned off fludrocortisone and desmopressin. A follow‐up evaluation by endocrinology ruled out diabetes insipidus (DI) and confirmed CSWS by evaluating repeat urine studies outlined in Table 3. The patient was later transferred out on salt tablets and discharged to a nursing facility in stable medical condition. In regard to the patient′s perspective, the overall experience was tumultuous. Although the patient was safely transferred to a nursing facility, he was hospitalized for several weeks and had to manage the unpleasant alarms, which were both constant and distressing.

**Table 3:** Pre and posttreatment urine studies.

**Urine osmolality**	**Sodium studies**
Pretreatment urine studies	
552 mOsm/kg	147 mmol/L
Posttreatment urine studies	
184 mOsm/kg	72 mmol/L

## 3. Discussion

CSWS is a rare etiology of hypovolemic hyponatremia in patients with central nervous system bleeding. As reflected in the medical literature, the presentation of CSWS is infrequent, especially in the setting of end‐stage heart failure. CSWS must be differentiated from SIADH, which can also be present in brain bleeds, yet the management is profoundly different. CSWS necessitates aggressive fluid hydration and sodium supplementation, whereas SIADH demands strict fluid restriction. Proper fluid management becomes crucial in LVAD patients, as they are preload‐dependent and afterload‐sensitive [7]. Dramatic volume shifts can directly impact how the LVAD functions. Furthermore, in the management of any patient with end‐stage heart failure, the traditional practice is to minimize fluid intake to avoid volume overload states. Initially, these two pillars of management seem contradictory; however, this case demonstrates that aggressive fluid supplementation can be successfully executed in an LVAD patient with end‐stage heart failure. At the onset of CSWS, the patient′s LVAD speed was set at 5800 RPM and power at 4.6 W. At the time, the pulsatility index was low at 1.1 with multiple low‐flow alarms. Upon discharge from the ICU and the treatment of CSWS, the patient′s LVAD speed was set at 6000 RPM and power at 4.2 W. The LVAD was able to provide 5.2 L/min of blood flow, and the pulsatility index was normal at 3.1. The LVAD was able to provide the patient with an appropriate amount of blood flow once the underlying etiology of his hypovolemia was treated.

Appropriate management begins, however, with differentiating CSWS and SIADH. In this patient′s case, CSWS and SIADH were most aptly differentiated by the urine studies. In CSWS, patients will present with decreased sodium concentration, increased urine sodium concentration, and increased urine output. SIADH presents with decreased serum sodium concentration and increased urine sodium concentration, but urine output is within a normal range. Our patient′s urine output increased to 4 L/day. Furthermore, because SIADH is an issue with antidiuretic hormone, there would be an expected elevation in arginine vasopressin hormone, which the patient did not have. An endocrinologist was also consulted for the consideration of DI in the differential. DI can also present with hypovolemia and increased urine output due to decreased antidiuretic hormone levels. The key difference is that patients with DI would also present with hypernatremia and decreased urine sodium concentrations. Because the patient was hyponatremic, DI was ruled out.

Another interesting aspect of this case is the diagnostic challenges of making the diagnosis of CSWS postcraniotomy. Hyponatremia is the hallmark electrolyte derangement that would clue a healthcare provider to CSWS when the urine output is acutely robust [8]. In the setting of an acute subdural hemorrhage, the neurointensivist will often target a higher sodium level, typically in the range of 140–155 mmol/L. Increased serum sodium levels will form an osmotic gradient that can draw water out of brain tissue. This decreases brain swelling and intracerebral pressures, as elevated pressures can worsen subdural hemorrhages. Maintenance fluid with normal saline was initiated after the hemorrhage, which likely prevented the serum sodium levels from decreasing dramatically. It was only when the maintenance fluids were discontinued, the hyponatremia persisted, and urine output increased exponentially that CSWS became considered. Hyponatremia is not only important in diagnosing CSWS or SIADH, but also in the management of LVAD patients with severe heart failure. One study identified 342 patients with heart failure who underwent LVAD implantation between 2008 and 2019. In a multivariate analysis, hyponatremia was associated with a markedly increased risk of all‐cause mortality and recurrent HF hospitalizations [9]. Thus, hyponatremia may also serve as a marker of prognosis in severe heart failure.

In the context of low‐flow alarm states with LVADs, it is also important to do a very concise evaluation, as there are other life‐threatening differentials to consider. Right ventricular failure, unstable arrhythmias, acute cannula malposition, gastrointestinal bleeding, cardiac tamponade causing obstructive shock, and new‐onset thrombosis can be other causes of low‐flow states that are deadly. Utilizing the pulsatility index in narrowing the differential is vital. Higher pulsatility index values can reveal increased preload or reduced afterload, whereas lower values may point toward increased afterload or reduced preload [10]. Utilizing point‐of‐care ultrasound to assess volume status can also aid in determining the etiology.

Lastly, when evaluating patients with CSWS, BNP may be useful as an indicator of treatment progression. BNP is a peptide hormone that is secreted in the ventricles of the heart as a reaction to increased ventricular blood volume. BNP is a static marker, primarily reflecting cardiac wall stress rather than rapid intravascular volume changes. Despite this, BNP could be applied to the management of CSWS. In this case report, the patient had markedly elevated levels of BNP compared to his baseline, despite a robust urine output. His BNP remained elevated until CSWS was adequately treated with fluid hydration and sodium supplementation. BNP is not only secreted by the heart but also by the brain. In the setting of subdural hematomas, increased intracranial pressure can drive the secretion of BNP, which directly inhibits sodium resorption and leads to hyponatremia [11]. Despite this connection, the relationship between CSWS and BNP is infrequently explored in the medical literature. One literature review evaluated 15 different studies and failed to establish a strong connection between elevated natriuretic peptide levels and the diagnosis of CSWS [12]. While there is no convincing evidence in the diagnosis of CSWS, BNP may be a reasonable surrogate marker for treatment progression. As seen in this patient, the decreasing levels of BNP were seen as the patient clinically improved. Further research must be conducted to confirm the connection.

## 4. Conclusion

In summary, this case illustrates the sensitivity of LVAD patients to hypovolemia and the clinical significance of differentiating CSWS from SIADH. The management of CSWS and SIADH is fundamentally distinct, yet both can present in the setting of increased intracranial pressure. LVADs are preload‐dependent and afterload‐sensitive, as sudden alterations in overall volume status can affect the device′s functionality. CSWS is an atypical cause of hyponatremia that presents with a decrease in serum sodium, an increase in urine sodium, low serum uric acid, and increased urine output. This study confirms that aggressive fluid hydration in a patient with an LVAD and end‐stage heart failure can be conducted safely. Failure to rapidly treat CSWS can lead to respiratory arrest, brain stem herniation, and even death.

## Ethics Statement

This study does not require the Institutional Review Board.

## Consent

Informed consent was obtained from all patients included in the study.

## Disclosure

Permission has been obtained for all reproduced or adapted materials. An earlier version of this manuscript was presented at the Society of Critical Care Medicine Annual Congress in February 2025 for an oral presentation.

## Conflicts of Interest

The authors declare no conflicts of interest.

## Funding

No funding was received for this manuscript.

## Data Availability

Data sharing does not apply to this article as no datasets were generated or analyzed during the current study.
